# Editorial: Tropical Plant Responses to Climate Change

**DOI:** 10.3390/ijms23137236

**Published:** 2022-06-29

**Authors:** Isabel Marques, Ana Ribeiro-Barros, José Cochicho Ramalho

**Affiliations:** 1 PlantStress & Biodiversity Lab., Centro de Estudos Florestais (CEF), Instituto Superior de Agronomia (ISA), Universidade de Lisboa (ULisboa), Tapada da Ajuda, 1349-017 Lisboa, Portugal; 2 PlantStress & Biodiversity Lab., Centro de Estudos Florestais (CEF), Instituto Superior de Agronomia (ISA), Universidade de Lisboa (ULisboa), Quinta do Marquês, Av. da Republica, 2784-505 Oeiras, Portugal

The climate crisis is pushing the planet’s tropical plants towards their limits [1,2]. Plants cannot move, so they must develop strategies to overcome abiotic stresses, such as drought, salinity, and extreme temperatures, that impose direct limitations on plant growth, fertility, and productivity [3,4]. This special edition brings recent signs of progress towards understanding the molecular mechanisms underlying the influence of cold, high temperatures, elevated CO_2_, and salinity in several tropical plants.

Lin et al. [5] identified and characterized the number of Late Embryogenesis Abundant proteins (LEAs) and abscisic acid-, stress-, and ripening-induced proteins (ASRs) in *Canavalia rosea* (Sw.) DC. (Fabaceae), a wild legume occurring on coastal dunes found in tropical and subtropical regions. CrLEAs were found to be widely distributed in the different chromosomes of *C. rosea*. The CrLEA/CrASR superfamily was involved in the adaptation of *C. rosea* to different habitats. Ten CrLEA/CrASR members showed differences in gene expression in response to salt/alkaline stress, high osmotic stress, or ABA treatment, helping to expand our understanding of the role of CrLEA/CrASR genes in natural ecological adaptability and responses to abiotic stresses.

Several studies have demonstrated that grafting vegetable crops onto tolerant rootstocks can improve global plant performance and final productivity under different abiotic stress conditions [6]. To test the role of hormones in this process, Gálvez et al. [7] studied rootstock-mediated growth and yield responses in pepper plants, either non-grafted or grafted onto three commercial rootstocks, which were subjected to moderate salinity stress. They showed that developmental and physiological responses of pepper plants were associated with changes in the hormonal balance that helped to improve crop productivity under salinity stress. These results are potentially important to improve tolerance to saline conditions, highlighting the need to balance hormonal traits.

As atmospheric [CO_2_] continues to rise to unprecedented levels, understanding its impact on plants is imperative to improve crop performance and sustainability under future climate conditions. Marques et al. [8] studied the transcriptional changes promoted by elevated CO_2_ in two major traded coffee species: the allopolyploid *Coffea arabica* L. cv. Icatu and its diploid parent, *C. canephora* Pierre ex A. Froehner cv. CL153. The functional characterization unveiled important differences in the expression of genes between the two genotypes, even though significant responses were absent, supporting the positive effects of eCO_2_ in maintaining the balance of most physiological functions in coffee plants.

This positive effect also helped to overcome the heat stress in coffee leaves studied by Marques et al., at least until 37 °C [9]. Transcriptomic changes showed that both species of coffee (*Coffea arabica* L. cv. Icatu and *C. canephora* Pierre ex A. Froehner cv. CL153) could endure higher temperatures (37 °C) than previously assumed although being strongly affected by high temperatures (42 °C). This was mostly felt in *C. arabica* cv. Icatu, where genes related to ribulose-bisphosphate carboxylase (RuBisCO) activity, chlorophyll a-b binding, and the reaction centers of photosystems I and II were down-regulated, especially under 42 °C, and regardless of elevated CO_2_. Discovering heat-resilient traits will therefore be essential to create the next generation of coffee cultivars adapted to the future climate conditions, while maintaining global productivity and the high quality of coffee beans.

The rise of global temperatures might also influence the mechanisms of species invasion. Cai et al. 2021 [10] explored the molecular mechanisms underlying the response of an invasive plant (*Sphagneticola trilobata* L.) to high temperatures. In comparison with its native congener, *S. calendulacea*, the invasive species showed a better resistance to high temperatures than *S. calendulacea*. There was no accumulation of ROS in *S. trilobata* at high temperatures, contrary to what was found in *S. calendulacea*. In contrast, under heat stress, the gene expression of antioxidant genes and the enzyme activity of antioxidants in *S. trilobata* remained high, suggesting a stronger ROS-scavenging ability. These results suggest that the gradual warming of global temperature might be beneficial to accelerate the invasion area of *S. trilobata*.

Finally, understanding the role of symbiotic interactions between host plants and their endophyte microorganisms in conferring plant resistance is also an important task to tackle the effects of climate change. In this context, Li et al. 2021 [11] evaluated the influence of the endophytic fungus *Piriformospora indica* on the cold resistance of *Musa acuminata* cv. Tianbaojiao. Under low temperatures, colonization by this fungus triggered ROS scavenging in the host plants, while increasing plant cell-protective enzyme activities and osmoprotectant contents, and the up-regulation of several cold-responsiveness genes in the leaves. The generation of ROS acts as a signal transduction molecule that regulates plant–microbial interactions, being essential for defense responses and adaption to stresses.

Overall, these wide studies showed the involvement of stress-responsive genes and/or activity of ROS scavenging enzymes to mediate regulation and abiotic stress resistance. Several genes have been highlighted in these studies that can be selected as candidate genes for future functional investigations to study plant adaption to extreme environments. It is important to further clarify the mechanisms involved in regulating ROS signaling pathways and the interplay with multiple abiotic stresses. This improved knowledge can be used to boost crop productivity or to incorporate the associated genes into the genetic background of elite cultivars to increase their resistance to abiotic stress.
